# Supplementary material for: Using explainable machine learning to characterise data drift and detect emergent health risks for emergency department admissions during COVID-19
Source: Sci Rep. 2021 Nov 26;11:23017. doi: 10.1038/s41598-021-02481-y (PMC8626460; doi:10.1038/s41598-021-02481-y)
Supplement: Supplementary file 1 — Supplementary Table 1. [file 41598_2021_2481_MOESM1_ESM.pdf]

Supplementary Material

Name	Encoding	Additional notes
30 day visit count	N/A	ED visits by patient in last 30 days
Hour of day	N/A	
Weekday	N/A	
Patient age	N/A	
Triage category	Target encoding	Manchester triage system score
Triage painscore	N/A	Alder Hey triage pain score
Respiration rate	N/A	Breaths per minute
SpO2 scale 1	N/A	Pulse oximetry (oxygen saturation)
Systolic blood pressure	N/A	
Pulse rate	N/A	
Temperature	N/A	
History of smoking	One-hot encoding	
Current smoker	One-hot encoding	
Hypertension	One-hot encoding	
Harmful use of alcohol	One-hot encoding	
Depression	One-hot encoding	
Type 2 diabetes	One-hot encoding	
Asthma	One-hot encoding	
Lives alone	One-hot encoding	
COPD	One-hot encoding	
Alcohol screening	One-hot encoding	
Harmful use of alcohol with dependence	One-hot encoding	
Osteoarthritis	One-hot encoding	
Hypercholesterolaemia	One-hot encoding	
Anxiety	One-hot encoding	
Atrial fibrillation	One-hot encoding	
Anaemia	One-hot encoding	
Obesity	One-hot encoding	
Chronic kidney disease	One-hot encoding	
Hypothyroidism	One-hot encoding	
Type 1 diabetes	One-hot encoding	
Number of comorbidities	N/A	Comorbidities for patient listed on previous ED visits.
Arrival mode	Target encoding	
Attendance complaint	Target encoding	Chief complaint taken at attendance
Triage complaint	Target encoding	Chief complaint taken at triage
Triage discriminator	Target encoding	Complaint additional notes taken at triage

Table 1. Full list of features used as input for the admissions model. For each feature, the type of encoding (if applicable) is noted along with any additional explanatory remarks. The one-hot encoded comorbidities are limited to the top 20 most common, however, the number of comorbidities counts all listed in the UHS database.
